# Discerning the differential molecular pathology of proliferative middle ear lesions using Raman spectroscopy

**DOI:** 10.1038/srep13305

**Published:** 2015-08-20

**Authors:** Rishikesh Pandey, Santosh Kumar Paidi, Jeon Woong Kang, Nicolas Spegazzini, Ramachandra Rao Dasari, Tulio Alberto Valdez, Ishan Barman

**Affiliations:** 1 Laser Biomedical Research Center, Massachusetts Institute of Technology, Cambridge, Massachusetts, 02139, USA; 2 Department of Mechanical Engineering, Johns Hopkins University, Baltimore, Maryland 21218, USA; 3 Otolaryngology, Head and Neck Surgery, University of Connecticut, 263 Farmington Ave, Farmington, Connecticut, 06030, USA; 4 Otolaryngology, Head and Neck Surgery, Connecticut Children’s Medical Center, 282 Washington St, Hartford, Connecticut, 06106, USA; 5 Department of Oncology, Johns Hopkins University, Baltimore, Maryland 21287, USA

## Abstract

Despite its widespread prevalence, middle ear pathology, especially the development of proliferative lesions, remains largely unexplored and poorly understood. Diagnostic evaluation is still predicated upon a high index of clinical suspicion on otoscopic examination of gross morphologic features. We report the first technique that has the potential to non-invasively identify two key lesions, namely cholesteatoma and myringosclerosis, by providing real-time information of differentially expressed molecules. In addition to revealing signatures consistent with the known pathobiology of these lesions, our observations provide the first evidence of the presence of carbonate- and silicate-substitutions in the calcium phosphate plaques found in myringosclerosis. Collectively, these results demonstrate the potential of Raman spectroscopy to not only provide new understanding of the etiology of these conditions by defining objective molecular markers but also aid in margin assessment to improve surgical outcome.

Morphologic recognition under white light otoscopic examination remains the gold standard for diagnoses of most middle ear pathological conditions. However, stratifications using morphology alone involve significant inter-observer variability and provide limited insight into a disease’s defining biochemistry^1^,^2^. Consequently, a common motif in middle ear pathology is the over-treatment of patients under the worst-case assumption^3^. The inability to biochemically define pathologies is particularly evident in the management of cholesteatoma and myringosclerosis that on gross inspection exhibit nearly identical features^4,^^5^. While cholesteatoma is characterized by keratinization of squamous epithelium and aggressive growth of the tissue in the middle ear and mastoid cavity, myringosclerosis is marked by the calcification and hyalinization in the tympanic membrane. The etiology of these pathological conditions (especially, myringosclerosis) is poorly understood with limited insights available into the possible association and co-existence of these two conditions. Yet, given the *a priori* expectation of abnormal tissue, accurate differentiation of these pathologies is as critical as distinction from uninvolved tissue.

From a clinical perspective, two particular issues remain unsolved: 1) the recurrent nature of cholesteatoma due to the difficulty in assuring a clear surgical margin during surgery and 2) the inability to predict which retraction pocket will develop into a cholesteatoma. In this milieu, molecular methods, which provide objective biomarkers for diagnoses, may permit disease detection prior to morphologic manifestations and allow for more sensitive pathological segmentation. Employing non-invasive sensing of the middle ear environment, molecular methods can report not only on the anatomy of the condition but also on its underlying physiology—possibly predicting its future behavior^6^. While we and others have recently employed multi-colored reflectance and autofluorescence imaging to visualize and characterize the middle ear lesions, the lack of molecular specificity of these approaches impedes definition of the underlying biochemistry^7^,^8^,^9^. The goal of this study is, therefore, to develop diagnostic classifiers that accurately differentiate the relevant pathologies by focusing on biologically relevant, specific discriminatory features.

Here we report a novel Raman spectroscopic detection platform for label-free, high throughput biochemical analyses of the middle ear pathology. The detection system is based on molecular profiling via analysis of vibrational modes that provide a “fingerprint” of the pathophysiological conditions^10^,^11^. Given the exquisite molecular specificity of this technique, the spectral markers may provide new routes to recognition of cell types within tissues as well as objective detection and grading that exceeds current capabilities^12^. Furthermore, we reasoned that the vibrational spectroscopic information could be directly employed to identify the calcifications prevalent in myringosclerosis due to their unique and strong Raman-active vibrational modes. Our hypothesis of calcification detection is supported by our prior observations of similar calcified microstructures in the breast, where the presence of such components embedded in the tissue matrix is an important indicator of underlying lesions^13^,^14^.

We investigate the feasibility of using Raman spectroscopy for identification of middle ear pathology via rapid scanning of the freshly excised middle ear specimen. This scanning platform, therefore, significantly alleviates the sampling limitations associated with rapid histological assessment. Our spectroscopic measurements reveal that, in addition to the presence of calcifications, myringosclerosis presents novel spectral markers that collectively can be used to construct an accurate decision algorithm. A model of silicate-substitution in the calcifications is proposed to explain these new spectral markers that represent an unreported biochemical moiety in middle ear pathology. We envision that the substantive advantages of Raman spectroscopy in terms of molecular specificity and its ability to couple to a fiber-probe for *in vivo* label-free acquisition will enable its ready translation to otolaryngology practice, thereby reducing the number of unwanted repeat surgeries and improving the quality of the patient life.

## Results and Discussion

**Figure 1:**
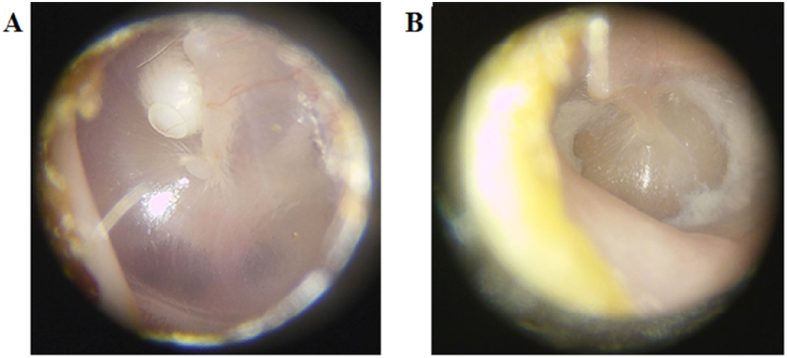
Representative white light images of (**A**) cholesteatoma and (**B**) myringosclerosis *in situ* prior to surgical excision of the lesions.

**Figure 2:**
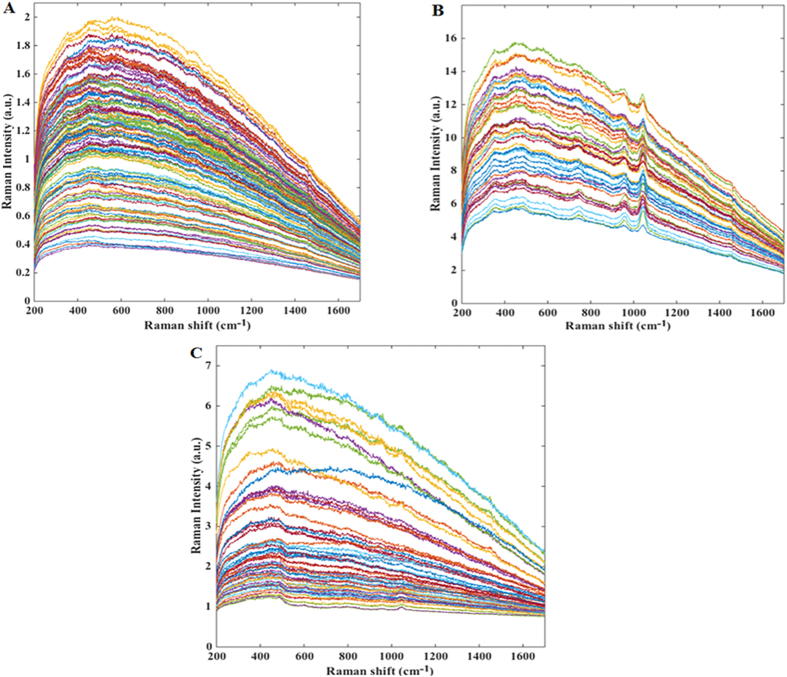
Representative Raman spectra acquired from: (**A**) cholesteatoma lesion sites, (**B**) myringosclerosis sites that exhibit mineralization on histological evaluation, and (**C**) myringosclerosis sites that display little or no mineralization (*i.e.* uninvolved tissue sites), respectively.

Figure 1 shows representative white light images of a cholesteatoma and a myringosclerosis lesion *in situ* before surgical excision. Figure 2 shows representative Raman spectra acquired from clinical tissue specimens post resection. The specificity of Raman spectroscopy in detecting molecular phenotypes of tissue reveals clear differences in the spectral signatures between the cholesteatoma lesions and some of the myringosclerosis sites. This is consistent with the medical consensus on these two pathological conditions, where the former is characterized by intrusions of keratinizing stratified squamous epithelium supported underneath by loose connective tissue (constituted largely by collagen and elastin) while the latter is comprised of calcified plaques amidst collagen deposits. Since the myringosclerosis lesions display significant heterogeneity in the spatial distribution of the calcified structures, treating the acquired spectral set from such tissue specimen as a homogeneous bucket would provide an inaccurate representation. Thus, based on the differences within the myringosclerosis set (particularly in the well-characterized Raman feature at 960 cm^−1^), we separated the mineralized sites (Fig. 2(B)) from the grossly uninvolved tissue (Fig. 2(C)) using peak identification code over a 20 cm^−1^ band centered at this feature. It is worth noting that the datasets show a measure of overlap that can be attributed to a continuous pathology model from uninvolved tissue to a site with high concentration of calcified structures. The differences, if any, between the cholesteatoma samples and the non-mineralized myringosclerosis set are more subtle and within-class variations in the spectral dataset impede the possibility of elucidating such differences by single-feature analysis alone.

**Figure 3:**
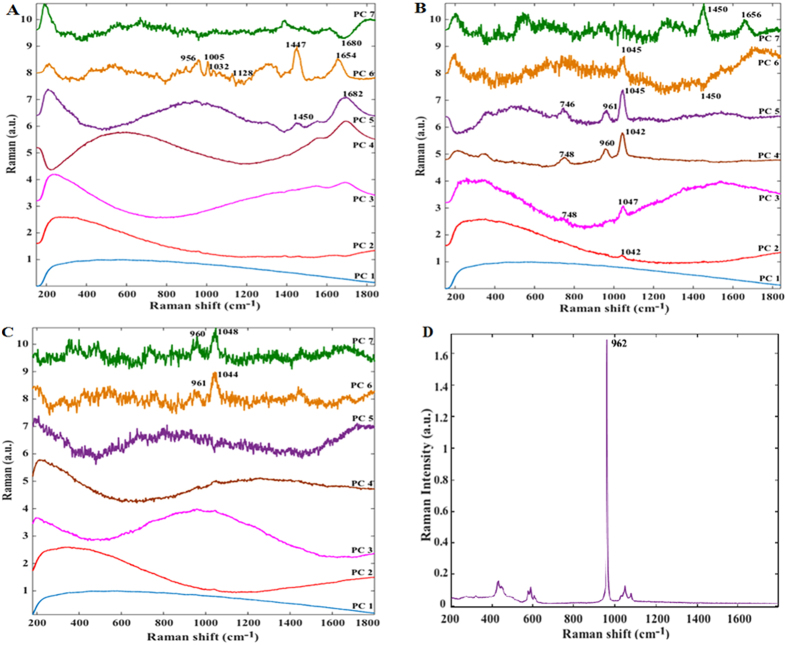
Illustration of principal component loadings for: (**A**) cholesteatoma lesion sites, (**B**) myringosclerosis sites that exhibit mineralization and (**C**) myringosclerosis with no appreciable mineralized clusters. Evidently, the PC loadings indicate the stark contrast in the underlying biochemistry between the different tissue pathologies. (**D**) Raman spectrum acquired from pure stoichiometric calcium hydroxyapatite for comparison.

Here we employed PCA to reduce the dimensionality of the spectral data into a few critical components that explain most of the data variance and to help identify “spectral markers” that can reliably discern the tissue pathology. Figure 3 shows the first 7 PCs for each of these three tissue types (cholesteatoma, biomineralized sites of myringosclerosis and uninvolved sites of myringosclerosis lesions) with the inset highlighting the Raman scattering features in the pertinent PC loadings. The broad autofluorescence background provides a significant contribution to the first few PCs, for each tissue type, despite the use of the NIR excitation source. While we have previously shown that the appropriate use of chemometric methods can aid in decoupling the autofluorescence signal from the Raman features of interest^15^, the shot noise associated with the autofluorescence background in the visible region can significantly impede the classification accuracy thereby highlighting the importance of working in the “tissue-transparent” NIR window.

The PC loadings for the cholesteatoma sites also exhibit Raman features of keratin and collagen as the main structural components of such lesions including 1005 cm^−1^ (C–C stretching vibration of the aromatic ring in the phenylalanine side chain), 1447 cm^−1^ (methylene, CH_2_, deformation band (scissoring)) and 1654 cm^−1^ (ν(C = O) stretching amide-I band) (Fig. 3(A)). Weaker features at 956 cm^−1^ (CH_2_ rock), 1032 cm^−1^ (C–H in-plane bending mode of phenylalanine) and 1128 cm^−1^ (skeletal C–C mode, trans conformation) are also visible. These observed features are in agreement with those reported in the literature, the detailed assignments and interpretations of which can be found elsewhere^16^,^17^,^18^. It is worth noting that some of these features can also be indicative of the presence of lipids (*e.g.* cholesterol and cholesterol ester), as postulated by Knudsen and co-workers^19^. Additionally, in relation to the amide-I vibration, we interestingly observe two potential features, one at 1654 cm^−1^ (α-helix) as previously noted and another at *ca.* 1680 cm^−1^, which suggests the existence of alternate conformations of the structural proteins in the lesion. This is not surprising given the continuous collagen degradation and bone resorption processes during the progression of this proliferative lesion^20^.

While gross inspection of the myringosclerosis lesions revealed some differences from cholesteatoma cases, PCA of the myringosclerosis sites with and without mineralization reveals dramatic differences in the underlying biochemistry. Outside of the broad autofluorescence background, the myringosclerosis sites consisting of mineralized clusters displays little in common with the aforementioned cholesteatoma features – even though both look nearly identical under white light otoscopic examination. In particular, PCs highlight an intense peak at *ca.* 1044 cm^−1^ with another strong peak at 960 cm^−1^ and a less intense feature at 748 cm^−1^ (Fig. 3(B)). Since the formation of calcium phosphate plaques in the lamina propria of the tympanic membrane is well-known in myringosclerosis, the presence of the 960 cm^−1^ peak, the *v*_1_(PO_4_) totally symmetric stretching mode of the “free” tetrahedral phosphate ion, is expected. Finally, PC loadings 6 and 7 also exhibit Raman features at 1447 and 1654 cm^−1^, albeit at much smaller intensities than for the aforementioned peaks observed in PCs 3-5, indicating the presence of loose connective tissue. On the other hand, Fig. 3(C) shows that the PC loadings corresponding to sites with little or no mineral components (as verified on histological examination) display noisier profiles. Nevertheless, the presence of the weak features at 960 and 1048 cm^−1^ in PC7 indicates that morphologically uninvolved tissue, particularly at lesion margins, may be biochemically distinct from normal tissue, *i.e.* molecular modifications in the margins could be the precursors of lesion development.

Of considerable interest is the presence of the 1044 cm^−1^ that has, thus far, not been identified in the literature in the context of middle ear pathogenesis. Given the intensity of these peaks, especially the 1044 cm^−1^ feature, one can reasonably infer that it emanates from a Raman-active mineralized constituent as opposed to the surrounding protein matrix. In fact, biological apatite is a poorly crystalline, non-stoichiometric material (Ca:P molar ratio < 1.67) that may contain additional ions in the structure such as Na^+^, SiO^4−^, CO_3_^2−^, Zn^2+^ and Mg^2+^ 21,22,23,24,25. By examination of the acquired spectra and its comparison with that of pure stoichiometric calcium hydroxyapatite (Fig. 3(D)), it is evident that the structures in these lesions are not composed solely of apatite. For example, there is considerable broadening of the 960 cm^−1^ band in the acquired spectra in relation to the sharp feature obtained from pure apatite to the extent that another phosphate *v*_1_ mode that occurs at 948 cm^−1^ is obscured by the broad phosphate stretching mode at 960 cm^−1^. An analogous finding of phosphate peak broadening has been reported in type II microcalcifications in breast tissue, where the introduction of carbonate ions into the apatite structures has been correlated with increasing malignancy of the lesion^13^,^26^.

We hypothesize that similar anionic substitutions is prevalent in the calcium phosphate plaques in myringosclerosis. The 1044 cm^−1^ peak can then be attributed to the presence of asymmetric stretching (*v*_3_) of the P–O bond observed in carbonate- and silicate-substituted phosphate, as detailed in Chaudhury *et al.*^27^, with the relative strength of this peak in relation to 960 cm^−1^ depending on degree of substitution. Furthermore, based on the absence of strong carbonate peaks at 912 and 1477 cm^−1^, we are of the view that the silicate-substitutions dominate the biomineralized constituents in these lesions. At this point, it is important to consider the possible origin(s) of such apatite structures and, critically, the presence of silicate-substitutions from a pathophysiological perspective. As Creusy and co-workers have noted, the most imperative condition for apatite formation is an exceedance of the critical supersaturation level by the component ions in the microscale milieu^28^. Here, the critical supersaturation signifies a value close to the solubility product beyond which the component ions of the crystal do not remain in solution but precipitate and form aggregates^28^. Cartilage fluids are marginally supersaturated with CaPO_4_, the principal component ion of biological apatite, but do not crystallize in physiological conditions due to the presence of various chelators and crystallization inhibitors^29^,^30^,^31^,^32^. However, a pathological condition such as chronic otitis media or the sudden insertion of a tympanostomy tube (grommet) could shift the supersaturation level towards a higher ionic disequilibrium, producing an environment that favors apatite formation. Additionally, emerging data from *in vitro* model studies of mammary cell mineralization suggest that the presence of hydroxyapatite crystals in the extracellular matrix could, in turn, enhance the proliferation of the lesion^33^.

Contrary to apatite, the biochemical origin and clinical relevance of silicate substitutions in the middle ear are elusive. We hypothesize that the formation of such structures is an end result of severely disrupted cellular homeostasis, a major determinant of which is the presence of silicone tympanostomy tubes. In terms of the impact, soluble silicate ions have been found to stimulate the expression of type-I collagen in osteoblast-like cell cultures^34^. Furthermore, *in vivo* assessments have demonstrated enhanced bioactivity of silicon-substituted hydroxyapatite over pure hydroxyapatite^34^ indicating that growth of such lesions could be faster than those in the presence of purely stoichiometric apatite.

**Figure 4:**
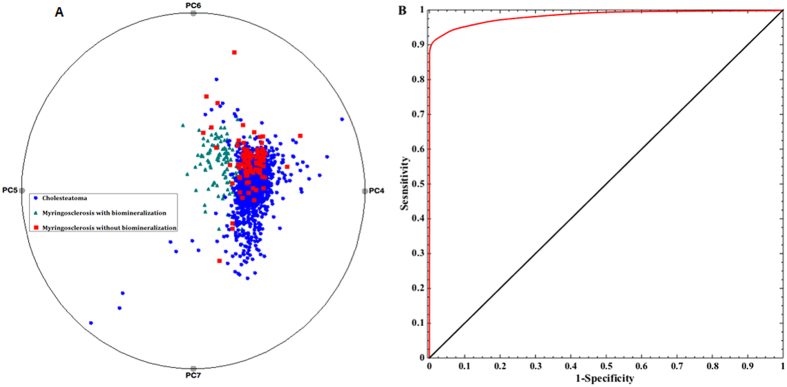
(**A**) Multi-dimensional radial visualization plot of selected principal component scores obtained from the entire spectral dataset. The plot illustrates the clustering behavior of the data points corresponding to the myringosclerosis sites that exhibit mineralization. (**B**) ROC curve for PLS-DA derived algorithm for the diagnosis of mineralized myringosclerosis sites. The ROC curve in red plots sensitivity versus (1-specificity) for the PLS-DA decision algorithm as the discrimination threshold is varied. For comparison, the ROC curve of two indistinguishable classes (represented by the solid black line) is also shown.

In addition to identifying a robust set of biologically relevant spectral markers, we also sought to develop a decision algorithm that could rapidly delineate the pathology of the tissue in a label-free, real-time manner. First, a nonlinear radial visualization map was constructed to plot the PC data dimensions onto a two dimensional space for the purpose of clustering (Fig. 4(A))^35^. These PC scores were extracted from the entire spectral dataset – and not from separate loadings as identified in Fig. 4. The PCs describing tissue site biochemical characteristics are equally spaced around the perimeter of a circle and provide dimension anchors, where the values of each dimension (PC score) are standardized between 0 and 1. Each tissue site is shown as a point inside the unit circle with its location governed by its dimension anchors. The radial visualization plot reveals an almost clearly separable cluster for the biomineralized myringosclerosis sites with substantial overlap between the cholesteatoma sites and the other myringosclerosis sites, stemming from the lack of distinctive spectral markers that separates the latter two. The few incorrectly segregated myringosclerosis sites can be attributed to the spectroscopy-histopathology registration error. It is worth noting that a high degree of reproducibility when replicate measurements were performed on the same sites in each tissue specimen. Expectedly, the inter-sample variations were larger than the intra-sample changes – which can be attributed to the intrinsic heterogeneity of tissue specimen acquired from different individuals.

To quantify the discrimination ability, PLS-DA decision algorithms were developed and tested in a leave-*n*-site-out cross-validation routine. First, a subset of cholesteatoma spectra were randomly chosen in order to constrain the number of data points for each class to be comparable. This was undertaken to prevent overtraining that would otherwise skew the prediction results. Second, 60% of the observations from each class were used for PLS-DA model training and the rest were used to constitute an independent test set. These two steps were each iterated 100 times each to get a robust estimate of the prediction capability. We computed the average correct classification rates to be *ca.* 73%, 95% and 76% for cholesteatoma, biomineralized myringosclerosis sites and other myringosclerosis sites without apatite content, respectively. As expected, the myringosclerosis sites showing biomineralization are classified with high accuracy owing to the presence of reliable spectral markers. The robustness of the PLS-DA derived decision algorithm was tested using a negative control study where arbitrary labels were assigned to the spectral data regardless of their true origins. In this case, the average correct rate of classification was found to be *ca.* 33% that can be attributed to random chance of picking one correct class out of three. The low value of classification accuracy in the control study confirms the robustness of the algorithm to spurious and chance correlations.

Finally, binary classification models were implemented using only the mineralized myringosclerosis and cholesteatoma data. This yielded positive predictive value (PPV) of 99.02%, negative predictive value (NPV) of 95.63%, sensitivity (SE) of 95.48%, specificity (SP) of 99.06%, and overall accuracy (OA) of 97.27%. When the same algorithm was applied to the mineralized myringosclerosis dataset against spectra from cholesteatoma and other myringosclerosis sites combined, the values of PPV, NPV, SE, SP and OA obtained were observed to be 90.32%, 95.81%, 92.29%, 94.68% and 93.85%, respectively. Figure 4(B) shows the corresponding receiver operating characteristic (ROC) curve (plot of sensitivity versus (1-specificity)), where area under the curve is computed to be 0.98 (for comparison, the AUC of a perfect algorithm is 1.00). The slight degradation of the performance due to merging the cholesteatoma sites with the non-mineralized myringosclerosis sites support our hypothesis of possible biochemical changes in the non-mineralized sites in the myringosclerosis tissue prior to their morphologic manifestations.

Although our pilot scale study does not warrant general biological conclusions, the segmentation achieved through a small subset of features reflects the ability of Raman spectroscopy in augmenting existing –omics technologies in providing biologically and clinically meaningful data. Our observations suggest a potentially important role of the silicate-substituted hydroxyapatite in the pathogenesis of myringosclerosis that warrants further investigation. Additional studies are ongoing to validate the identified molecular signatures in a larger cohort of patients.

The present study provides the first application of Raman spectroscopy in diagnosis and differentiation of cholesteatoma and myringosclerosis. Identification of the spectral markers (molecular targets) from spatially localized regions offer much-desired quantifiable data to enable early detection and longitudinal monitoring of middle ear pathology not only to improve patient management, but also to serve as a medical research tool to better understand the fundamental processes of lesion formation. In this work, we have reported the first evidence of the presence of carbonate- and silicate-substitutions in the calcium phosphate plaques found in myringosclerosis. Our efforts here represent the first step toward the deployment of vibrational spectroscopy as a label-free, real-time and molecular specific probe for the guidance of middle ear surgical procedures. Current *in vivo* studies involving larger patient cohorts at Connecticut Children’s Medical Center will be used to test the hypothesis that spectroscopic measurements can reliably identify residual disease thereby enhancing surgical removal and improving patient prognosis.

## Methods

### Human subject studies

The protocol of the present study was approved by the Institutional Review Board at Connecticut Children’s Medical Center. Inclusion of patients in this pilot study was limited to those undergoing an otologic surgical procedure under general anesthesia. Informed patient consent was obtained before the study. In this pilot study, samples were surgically removed from six patients after white light otoscopic examination by an experienced otolaryngologist (T.A.V.). The set of unfixed, de-identified tissue specimens – grossly indicative of cholesteatoma or myringosclerosis lesions respectively – were shipped frozen on dry ice and thawed at room temperature before scanning. To prevent dehydration, the tissues were moistened with a small amount of normal saline. The tissues were placed side-by-side on the scanning platform for Raman spectral acquisition as detailed below. After spectral collection, the samples were fixed in 10% neutral buffered formalin and paraffin-embedded, sectioned and stained for histopathological analysis. All experiments were performed in accordance with the approved guidelines and regulations.

### Raman spectroscopic measurements

The samples were placed on quartz cover slips to enable scanning measurements in an inverted geometry and to reduce substrate interference. In order to assess the feasibility of high-throughput measurements, we performed the experiments on a home-built a fiber probe-based flatbed scanner at the MIT Laser Biomedical Research Center. Based on the original design of a diffuse reflectance and autofluorescence scanner we had previously developed^36^, wide area spectroscopic imaging was achieved by mechanically scanning the beam. Spectral recording time was 100 msec/pixel. Here, a 785 nm compact solid-state laser is used as excitation source and the collected light is recorded on a spectrograph (Holospec f/1.8i, Kaiser Optical Systems) and a thermoelectric-cooled, back-illuminated, and deep depleted CCD (PIXIS: 100BR_eXcelon, Princeton Instruments). The power at the sample was held constant at 70 mW and the spectral recording time was 100 msec/pixel. The recorded spectra were corrected for the presence of cosmic rays before further data analysis and interpretation.

### Data analysis

The Raman system was wavenumber calibrated and corrected for the system wavelength response and fiber probe background. The spectral dataset obtained using the scanning platform contained the spectra from tissue and substrate (where tissue was absent) in the area scanned. Due to the possibility of introducing spectral artifacts, no further processing (e.g. removal of the autofluorescence background/baseline correction) was undertaken. To visualize the ability of Raman spectroscopic measurements to differentiate between the pathophysiological sites, principal component analysis (PCA) was employed on the entire tissue spectral dataset using the Statistics Toolbox of MATLAB R2014b (Math Works, Natick, MA). PCA is a widely used data exploration technique and is extensively employed to understand the clustering (or the lack thereof) of high-dimensional spectroscopic data. Radviz, a component of Orange data mining software^37^, was subsequently used to plot the scores against a set of selectively chosen principal components (PCs) for optimal visualization of the class separation. Finally, partial least squares-discriminant analysis (PLS-DA) was employed to build decision algorithms to quantify the segmentation capability.

## Additional Information

**How to cite this article**: Pandey, R. *et al.* Discerning the differential molecular pathology of proliferative middle ear lesions using Raman spectroscopy. *Sci. Rep.* **5**, 13305; doi: 10.1038/srep13305 (2015).
